# Autoantibody Diagnostics in Neuroimmunology: Experience From the 2018 Italian Neuroimmunology Association External Quality Assessment Program

**DOI:** 10.3389/fneur.2019.01385

**Published:** 2020-01-14

**Authors:** Matteo Gastaldi, Elisabetta Zardini, Silvia Scaranzin, Antonio Uccelli, Francesca Andreetta, Fulvio Baggi, Diego Franciotta

**Affiliations:** ^1^Neuroimmunology Laboratory, IRCCS Mondino Foundation, Pavia, Italy; ^2^Department of Brain and Behavioral Science, University of Pavia, Pavia, Italy; ^3^Department of Neuroscience, Rehabilitation, Ophthalmology, Genetics, Maternal and Child Health (DINOGMI), University of Genoa, Genoa, Italy; ^4^Ospedale Policlinico San Martino - IRCCS, Genoa, Italy; ^5^UO Neurology IV, IRCCS Fondazione Istituto Neurologico Carlo Besta, Milan, Italy

**Keywords:** external quality assessment scheme, standardization, neuroimmunology, antibodies, tissue-based assays, cell-based assays, radioimmunoassays, ELISA

## Abstract

**Background:** Neuroimmunology has impressively expanded in the past decade. Novel assays, especially cell-based assays (CBAs) can detect conformational antibodies (Abs) recognizing antigens in their native conformation. Generally, the availability of in-house and of commercial tests has improved the diagnostics, but introduced demanding laboratory tasks. Hence, standardization and quality controls represent a key step to promote accuracy. We report on the results of the 2018 external quality assessment program (EQAP) organized by the Italian Neuroimmunology Association.

**Methods:** EQAP regarded 10 schemes, including oligoclonal bands (OCBs), intracellular-neuronal (ICN)-Abs, neuronal-surface (NS)-Abs, aquaporin-4 (AQP4)-Abs, myelin oligodendrocyte glycoprotein (MOG)-Abs, myelin-associated glycoprotein (MAG)-Abs, ganglioside-Abs, acetylcholine-receptor (AChR)-Abs, and muscle-specific-kinase (MuSK)-Abs, and 34 laboratories. Assays were classified as tissue-based assays (TBAs), solid-phase assays (SPAs), liquid-phase assays (LPAs), and CBAs. Thirty-three samples were provided.

**Results:** Three-quarter of the tests were commercial. Median accuracy for the laboratories was 75% (range 50–100). In 8/10 schemes, at least one sample provided discrepant results. Inter-laboratory “substantial agreement” was found in 6/10 schemes (AChR, MuSK, MAG, AQP4, MOG, and NS-Abs), whereas the worst agreements regarded OCBs and ganglioside-Abs. Both commercial and in-house assays performed better in experienced laboratories.

**Conclusions:** Assays could be divided in (a) robust commercial tests with substantial inter-laboratory agreement (MAG-Abs; AChR- and MuSK-Abs); commercial/“in-house” tests with (b) partial inter-laboratory agreement (AQP4-Abs, MOG-Abs, NS-Abs, ICN-Abs), and (c) with large inter-laboratory disagreement (OCBs, ganglioside-Abs). This real-life snapshot of the neuroimmunology test performances highlights shortcomings attributable to technician-dependent performances, assay structural limitations, and errors in test interpretations.

## Introduction

External quality assessment (EQA) testing is part of a wider educational approach aimed to improve and monitor quality in laboratory diagnostics. Since 2000, the Italian Association of Neuroimmunology (AINI) has espoused this commitment, which includes the production of standardizations of methods and of clinic-laboratory guidelines (1). Over these years, neuroimmunology diagnostics has been facing formidable challenges, especially after the discovery of autoantibodies to cell-surface neuroglial proteins, which associate with many potentially treatable neurological disorders (2, 3). Such autoantibodies preferentially bind antigens when their tertiary structure is preserved. This has revolutionized the neuroimmunology diagnostics, with the diffusion of “conformational” tests, such as cell-based assays (CBAs) and immunohistochemistry on lightly-fixed brain tissues for the diagnosis of autoimmune encephalitis (4), and for the differential diagnosis of the acquired demyelinating diseases of the CNS, including multiple sclerosis (5).

These new techniques have been developed as in-house protocols in specialized laboratories, thus requiring a proper expertise that often lacks in the large clinical chemistry laboratories using commercially available CBAs. In these laboratories, moreover, neuroimmunology diagnostics performed with automated or semi-automated systems is increasingly incorporated.

We herein report on the results of the 2018 EQA program that involved Italian laboratories of the AINI network, and that was extended to few European laboratories. These results provide a snapshot on how the participating laboratories perform, and useful information on the degree of reliability and accuracy characterizing each single test in real life.

## Materials and Methods

### External Quality Assessment Program Design

The Neuroimmunology Laboratories in Pavia and in Milan were the program coordinators. The program was composed of 10 schemes, each addressing different areas of neuroimmunology diagnostics: oligoclonal IgG bands (OCBs) detection [with isoelectric focusing (IEF)] and pattern interpretation, intracellular neuronal antibodies (ICN-Abs), neuronal surface antibodies (NS-Abs), aquaporin-4 antibodies (AQP4-Abs), myelin oligodendrocyte glycoprotein antibodies (MOG-Abs), myelin associated glycoprotein antibodies (MAG-Abs), ganglioside-Abs, acetylcholine receptor antibodies (AChR-Abs), and muscle specific kinase antibodies (MuSK-Abs). Twenty-nine Italian and five European laboratories participated to the EQA program (Supplementary Table 1 and Supplementary Figure 1). Each laboratory chose to take part to any number of the proposed schemes. The procedures for sample handling are described in Supplementary Figure 2.

A total number of 25 serum samples and 4 serum-CSF pairs were used (Table 1). The clinical diagnosis associated to each sample was established by trained neurologists (MG, DF, and FB). The results obtained by the coordinating centers (Pavia and Milan) were considered as the reference results. The participating laboratories were requested to test the samples according to their own routine standard operating procedures, and results were reported to the coordinating team using a result form. Report forms asked to classify the tested sample as “positive” or “negative” and to report the specific antibody type detected. Quantitative results from enzyme-linked immunosorbent assay (ELISA) and radioimmunoassays (RIAs) were collected, when appropriate.

**Table 1:** Samples used in the AINI EQA program.

**Test**	**Sample N**	**Code**	**Material**	Titer^*^	**Clinical Diagnosis**	**Sent as**
Isolectric focusing	1	S1L1	Serum-CSF pair	–	Post-infectious encephalomyelitis	Mirror (pattern #4)
	2	S2L2	Serum-CSF pair	–	Hydrocephalus and MGUS	Monclonal gammopathy (pattern#5)
	3	S3L3	Serum-CSF pair	–	Multiple Sclerosis	Mixed (pattern#3)
	4	S4L4	Serum-CSF pair	–	Clinically Isolated Syndrome	OCB (pattern#2)
Onconeural antibodies	5	O1	Serum	NA	Paraneoplastic cerebellar degeneration (ovarian tumor)	Yo pos
	6	O2	Serum	NA	Stiff person syndrome	GAD pos
	7	O3	Serum	NA	Healthy control	Neg
Neuronal Surface antibodies	8	C1	Serum	1:1200	Limbic encephalitis	LGI1 pos
	9	C2	Serum	1:400	NMDAR encephalitis	NMDAR pos
	10	C3	Serum	–	Healthy control	Neg
AQP4 antibodies	11	Q1	Serum	–	Healthy control	Neg
	12	Q2	Serum	1:10	NMOSD	Pos
	13	Q3	Serum	1:100	NMOSD	Pos
MOG antibodies	14	G1	Serum	1:160	Optic neuritis	Pos
	15	G2	Serum	–	Healthy control	Neg
	16	G3	Serum	1:640	Transverse myelitis	Pos
MAG antibodies	17	MAG1	Serum	40000_BTU_	DADS neuropathy	Pos
	18	MAG2	Serum	25000_BTU_	DADS neuropathy	Pos
	19	MAG3	Serum	17000_BTU_	DADS neuropathy	Pos
Ganglioside antibodies	20	P1	Serum	NA	Miller-Fisher syndrome	Gq1b IgG pos
	21	P2	Serum	–	Healthy control	Neg
	22	P3	Serum	NA	CANOMAD	GD1b IgM and GQ1b IgM pos
	23	P4	Serum	NA	Motor Multifocal Neuropathy	GM1 IgM pos
AChR antibodies	24	A1	Serum	3.2 nmol/L	Myasthenia gravis	Pos
	25	A2	Serum	7.8 nmol/L	Myasthenia gravis	Pos
	26	A3	Serum	–	Healthy control	Neg
MuSK antibodies	27	M1	Serum	1.2 nmol/L	Myasthenia gravis	Pos
	28	M2	Serum	–	Healthy control	Neg
	29	M3	Serum	1.4 nmol/L	Myasthenia gravis	Pos

*CSF, cerebrospinal fluid; MGUS, monoclonal gammopathy of uncertain significance; OCB, oligoclonal bands; NA, not available; GAD, glutamic acid decarboxylase; LGI1, leucine rich glioma inactivated protein 1; NMDAR, N-methyl-D-aspartate receptor; AQP4, aquaporin 4; NMOSD, neuromyelitis optica spectrum disorder; MOG, myelin oligodendrocyte glycoprotein; MAG, myelin associated glycoprotein; BTU, Bühlmann Titer Units; DADS, distally acquired demyelinating sensory neuropathy; CANOMAD, Chronic Ataxic Neuropathy, Ophthalmoplegia, IgM paraprotein, cold Agglutinin, Disialosyl antibodies; AChR, acetylcholine receptor; MuSK, muscle specific kinase*.

* *Titres are reported as endpoint titrations unless otherwise specified according to the coordinating centers results*.

All the results of the present EQA program will be presented anonymized, to preserve the confidential nature of the single laboratory performance.

### Assays

Assays were classified as: (a) solid-phase assays (SPAs), including blots and ELISA; (b) tissue-based assays (TBAs), including immunohistochemistry/immunofluorescence on rodent and primate brain, or peripheral nerve; (c) cell-based assays (CBAs), including live and fixed CBA; (d) liquid-phase assays (LPAs), namely RIAs.

Commercial assays were performed according to manufacturer's instructions. In house CBAs and TBAs were performed according to published protocols, but adapted to each laboratory routine (6–10).

### Statistical Analysis

Test results were considered as “concordant” or “discordant” when they matched/did not match the reference result, and “partially concordant” when they either reported incompletely what provided as reference, or when an additional positivity not included in the reference result was reported.

Qualitative variables were summarized as percentages, and quantitative variables as median with ranges.

Accuracy was calculated for each laboratory (frequency of tests concordant with the reference result among all the tests performed by the single laboratory). Between-laboratory agreement for each scheme was calculated using Fleiss' Kappa test with 95% confidence intervals (CI). Agreement was classified as following: “poor,” kappa = 0.0; “slight,” 0.00 ≤ kappa ≤ 0.20; “fair,” 0.21 ≤ kappa ≤ 0.40; “moderate,” 0.41 ≤ kappa ≤ 0.60; “substantial,” 0.61 ≤ kappa ≤ 0.80; “almost perfect,” 0.81 ≤ kappa ≤ 1.00 (11).

## Results

### Overall Results

Twelve/34 laboratories participating to the EQA program took part to 1–2 schemes, 10/34 to 3–5 schemes and 12/24 to >5 schemes (Supplementary Figure 1 and Table 1). The OCB scheme was the most attended (24/34 laboratories), followed by AQP4-Abs (20/34 laboratories).

**Figure 1:**
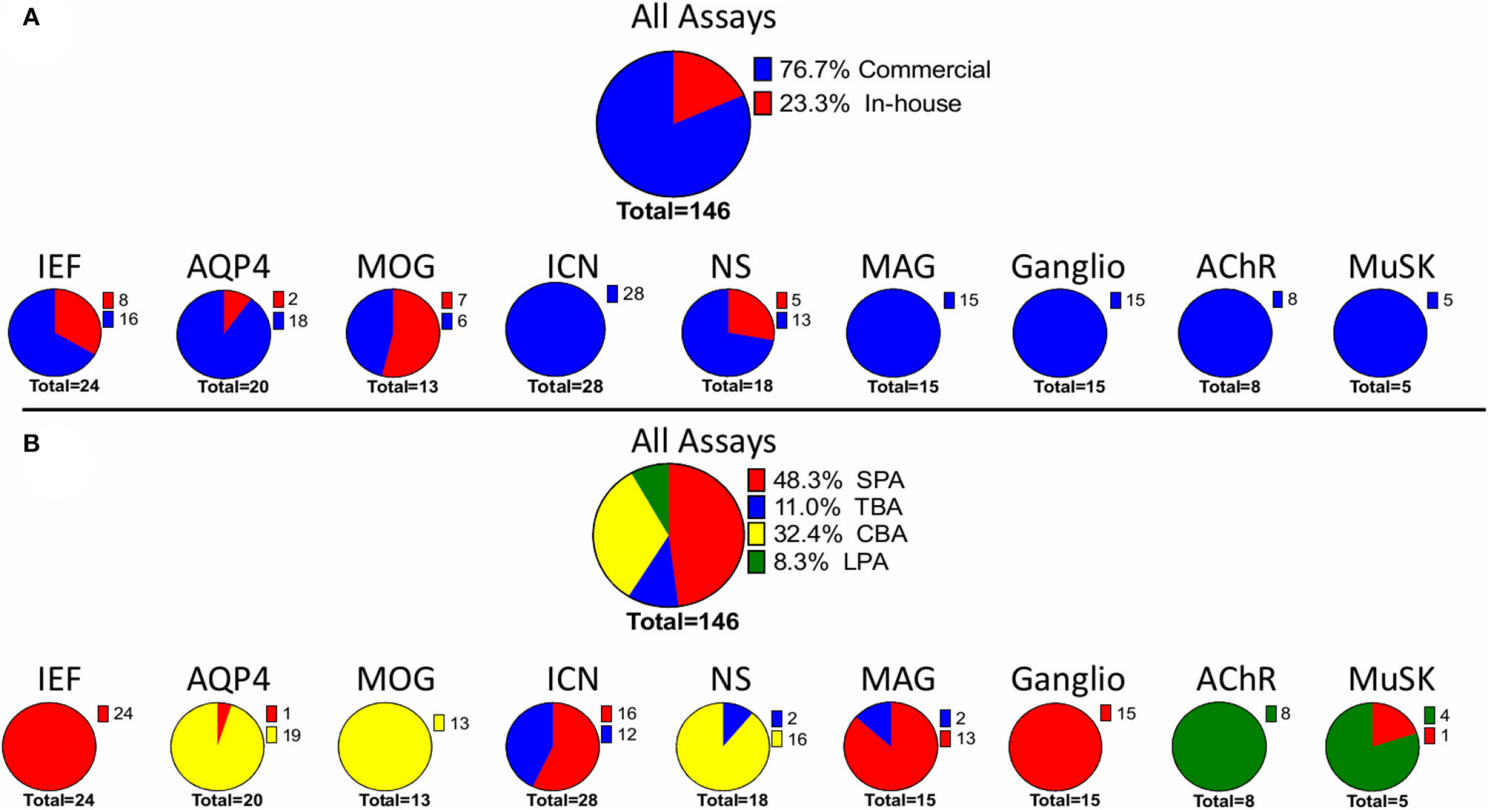
Assays used in the AINI-EQA program. The figure considers the number laboratory using **(A)** either in house or commercial assays or **(B)** a specific assay type. A single laboratory could use more than one assay. MOG, myelin oligodendrocyte glycoprotein; NS, neuronal surface; AQP4, aquaporin 4; MuSK, muscle specific kinase; ICN, intracellular neuronal; IEF, isoelectric focusing. SPA, solid phase assay; CBA, cell-based assay; LPA, liquid-phase assay; TBA, tissue-based assay.

Considering the total number of assays used by each laboratory in the EQA program, the most common assay type was SPAs (48.3%), followed by CBAs (32.4%) (Figure 1). Commercial assays were more common, and accounted for 76.7% of the total. The remaining 23.3% of the assays were made “in-house” (Figure 1).

**Figure 2:**
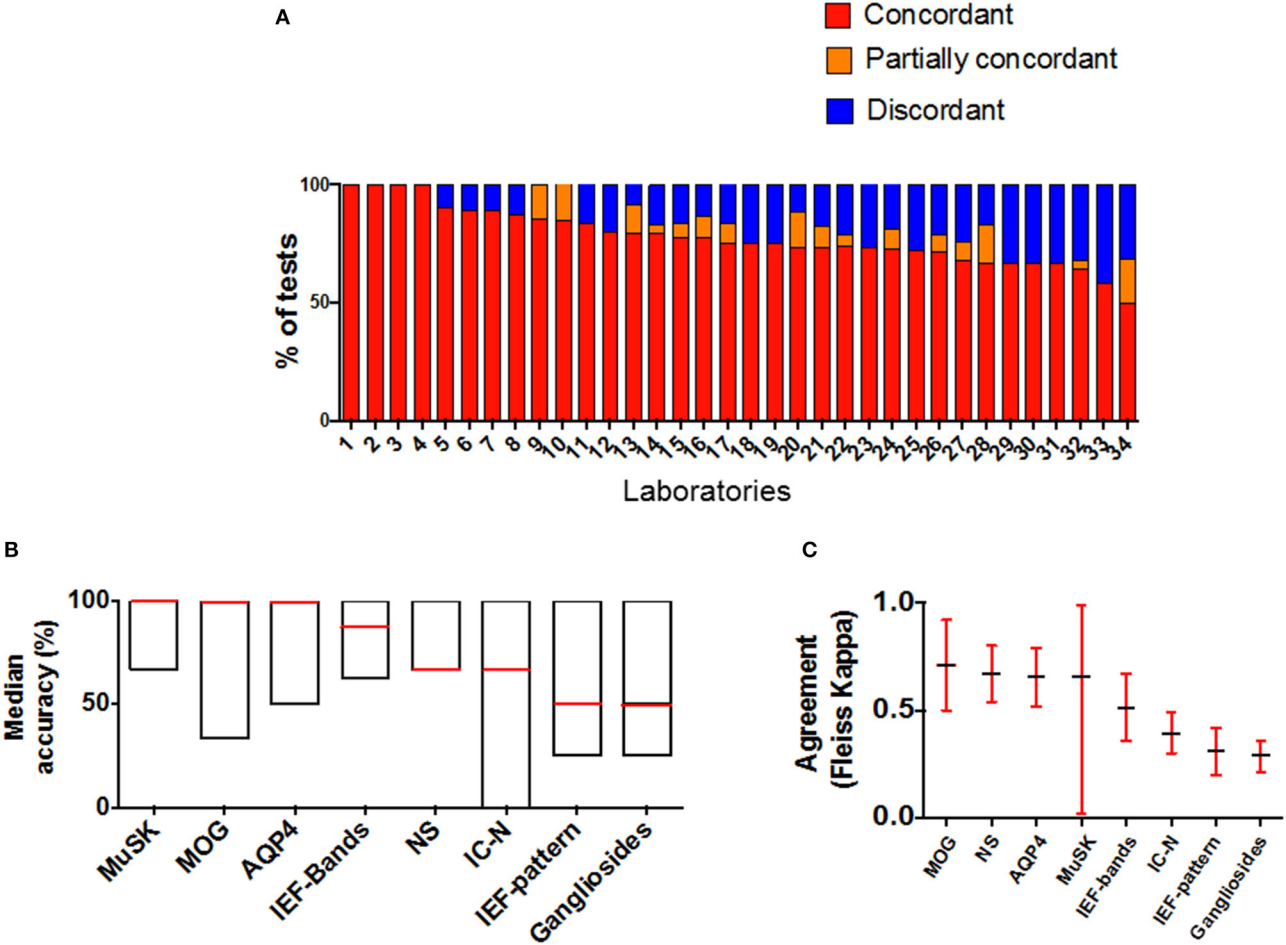
Laboratory performances and schemes results in AINI-EQA program. **(A)** Accuracy is represented by the number of concordant results obtained by each lab in all the schemes joined. Each lab participated to a variable number of schemes; **(B,C)** represent the performance **(B)** and the concordance of results **(C)** in each scheme (MAG and AChR schemes are not represented since all results were concordant). MOG, myelin oligodendrocyte glycoprotein; NS, neuronal surface; AQP4, aquaporin 4; MuSK, muscle specific kinase; ICN, intracellular neuronal; IEF, isoelectric focusing.

The overall performance of all laboratories is showed in Figure 2. Twelve/34 (67.6%) laboratories had an accuracy >80% (Figure 2A). Overall median accuracy was 75% (range 50–100) (Figure 2B; Supplementary Table 2).

In 8/10 schemes at least one sample was critical, providing at least one discordant result among laboratories. The highest number of discordant results was found in OCB pattern interpretation (39.6%), ICN-Abs (23.4%), and NS-Abs (23.4%) (Figure 2A). A “substantial agreement” between laboratories was found in 6/10 schemes.

Detailed results from each scheme are depicted in Supplementary Figures 3–5.

### Oligoclonal IgG Bands

#### Background of the Assay

The detection of the intrathecal production of oligoclonal immunoglobulins, which can be revealed in form of “discrete bands” on IEF, has high diagnostic relevance in multiple sclerosis (12), and in other inflammatory neurological diseases (13). Difficult-to-control factors, such as room temperature and humidity, gel conductivity, electroendosmosis, and ampholytes lot-to-lot differences, can affect the IEF technique making between-laboratory agreements very difficult to achieve (13). Interpretative issues of the IEF runs add complexity to the picture (14, 15). The introduction of semi-automated systems for IEF has simplified the test, but there is no comparison study on test performance vs. “in-house” assembled systems.

#### Results of AINI EQAS

The IEF scheme was split in two separate tasks. The first one required to establish presence or absence of OCBs in each of the four paired serum and CSF controls (8 samples), whilst the second required to interpret each of the ensuing IEF run as a whole, on the basis of the five patterns established by the 1994 consensus report on the topic (14).

S4L4 was the most critical sample, as it showed a few faint unique-to-CSF bands.

In this sample, bands were identified by 6/24 laboratories, and only 2/24 provided a correct interpretation of pattern #2 (14).

The serum-CSF pair S2L2, sent as pattern #5 (monoclonal gammopathy), was misinterpreted as a mirror, or a mixed pattern, by 10/24 laboratories.

As for the methods, 8/24 laboratories used “in-house” assembled IEF systems (where optimal run conditions were established in each laboratory), six using home-made agarose gels, two using commercial gels; the other 16 laboratories exploited semi-automated IEF systems. Overall accuracy of “in-house” assembled systems, which were used in the most experienced laboratories, was slightly, but not statistically significant superior than that of semi-automated systems, in both the task of band detection (85.9 and 81.3%, respectively), and of pattern interpretation (62.5 and 48.4%, respectively)

Overall agreement was “moderate” for bands detection (Fleiss' kappa = 0.51), and only “fair” for pattern interpretation (Fleiss' kappa = 0.31) (Figure 2C).

#### Conclusions

In line with the other previous programs on OCBs promoted by AINI, this EQA revealed the difficulties in detecting OCBs in critical samples. Even when recognized, OCBs can be misinterpreted as wrong patterns, with risks of wrong messages to the clinicians.

### AQP4 Antibodies

#### Background of the Assay

The presence of serum AQP4-Abs identifies acquired demyelinating syndromes of the CNS mainly affecting the optic nerves and spinal cord, collectively defined as Neuromyelitis Optica Spectrum Disorders (NMOSD) (16), which are in differential diagnosis with MS. Initially, AQP4-Abs were detected with immunohistochemistry on rodent brain, but currently CBAs are the gold standard (17, 18). When compared with ELISAs, CBAs offer the advantage of being conformational (19). The AQP4 protein arranges on the cell surface in tetramers, associated in the orthogonal particle arrays (OPAs) that are relevant for AQP4-Ab binding (20–22). In a multicenter comparison of AQP4-Abs detection assays, CBAs resulted the most sensitive assays (6). Both live and fixed CBAs showed good analytical performances, although live CBAs performed with slightly higher accuracy (6). The use of ELISAs is progressively decreasing due to inferior performances compared to CBAs (6, 17, 21).

#### Results of AINI EQAS

CBAs were used by 18/19 laboratories (“in-house” live CBAs for two of them), and only one used a commercial ELISA. The overall agreement was “substantial” (Fleiss' kappa: 0.66, 95%CI: 0.52–0.79). Fifteen/20 laboratories reached 100% accuracy. Sample Q2, a low AQP4-Ab-positive serum from an NMOSD patient (titer 1:10 on the commercial CBA) was reported as negative by 5/19 laboratories. Only one laboratory, using the commercial CBA, reported the reference negative sample Q3 as AQP4-Ab positive.

#### Conclusions

The interpretation of fluorescence in samples with low titers of AQP4-Abs can be challenging, and could lead to false negative results in the routine practice. The comparison between in-house and commercial CBA performances suggests that erroneous output evaluations mainly explained the relatively low concordance.

### MOG Antibodies

#### Background of the Assay

Using non-conformational methods, MOG-Abs had been associated with MS for decades (23). Subsequently, these antibodies, when detected with appropriate conformational methods, have been increasingly associated with non-MS acquired demyelinating syndromes, such as optic neuritis and transverse myelitis (7, 24–26). Since only conformational MOG-Abs are considered clinically relevant, CBAs are the gold standard for their detection (27). CBAs are performed on live cells transfected with human full-length MOG; bound IgG can then be detected with either an anti-total human-IgG (9), or an anti-human-IgG_1_, as secondary antibodies. (7) The output readout can be performed either by fluorescence microscopy, or flow-cytometry (28, 29). Recently, a commercial CBA for MOG-Abs detection relying on fixed cells has become available. In a three-center comparison, the fixed CBA showed rather good concordance with the live CBAs, with slightly lower specificity (30).

#### Results of AINI EQAS

Given the recent identification of MOG-Abs, this was the first year that the scheme was included in the AINI EQA program. Only laboratories using CBAs participated to this scheme, seven with “in-house” protocols with different characteristics of the secondary antibodies, which recognized total IgG (*n* = 3), IgG_1_ (*n* = 1), or both (*n* = 1). The remaining six laboratories used the commercial fixed CBA.

The two positive samples had medium to high titers (1:320–1:640), and were positive for IgG_1_ antibodies. The overall agreement was substantial (Fleiss' kappa: 0.71, 95%CI: 0.5–0.92). Eleven/13 laboratories correctly identified MOG-Abs in sample G1 and G2, and 13/13 recognized G3 as negative.

#### Conclusions

The participation of experienced laboratories only to this EQAS, using both live and/or fixed CBAs, likely accounted for overall good performances.

### Neuronal Surface Antibodies

#### Background of the Assay

NS-Abs represent an expanding group of autoantibodies targeting key proteins implicated in synaptic function (3, 31). These antibodies associate with a wide spectrum of disorders variably presenting with cognitive impairment, seizures, movement disorders, and autonomic dysfunction, defined as “autoimmune encephalitis” (2, 32). After the identification of antibodies against the N-methyl-D-aspartate receptor (NMDAR), many other NS-Abs have been discovered in the last years (33, 34), including those against leucine rich glioma inactivated-1 (LGI1) and contactin-associated protein-like 2 (CASPR2), α-amino-3-hydroxy-5-methyl-4-isoxazolepropionic acid receptor 1 and 2 (AMPAR), and γ-aminobutyric acid A or B receptor (GABA_A/B_R).

The use of conformational assays is crucial for NS-Ab detection (35), and includes CBAs and/or TBAs on rodent brain optimized with light fixation procedures (4). TBAs can be, at least for some Abs, more sensitive than CBAs, although CBAs are necessary to identify antigenic targets (36). The combination of TBAs and CBAs can improve diagnostic accuracy (37).

A commercial fixed CBA is currently available for the most frequently detectable NS-Abs. Rarer NS-Ab reactivities require appropriate “in-house” diagnostics (2).

#### Results of AINI EQAS

As most laboratories used the commercial test that includes only the most frequent NS-Abs (NMDAR-Abs, LGI1-Abs, CASPR2-Abs, AMPAR-Abs, and GABA_B_R-Abs), the EQA scheme was restricted to these Abs. Eleven/fourteen laboratories used the commercial CBA, whilst two used a strategy combining “in-house” TBAs and “in-house,” or commercial CBA. One laboratory used “in-house” live CBAs only (Table 2).

**Table 2:** Assays used in the AINI EQA program.

**Test**	**Assay**	N of labs/total^*^	**Main features**
Oligoclonal IgG bands	Semi-automated systems	15/23	Precast agarose gels (small-medium size); manufacturer's recommended run conditions; direct immunofixation
	In-house assembled systems	8/23	In-house or commercial precast agarose gels (large size); run conditions optimized in each laboratory; capillary blotting and immunofixation
Intracellular neuronal antibodies	Immunohistochemistry on fixed primate brain + blot A	6/16	Commercial (Euroimmun) chip + line-blot (Ravo), antigens: HuD, Yo, Ri, CV2 (CRMP5), Amphiphysin, Ma1, Ma2
	Immunohistochemistry on fixed primate brain + blot B	6/16	Commercial (Euroimmun) chip + line-blot (Euroimmun), antigens: HuD, Yo, Ri, CV2 (CRMP5), Amphiphysin, Ma, PCA-2, Tr, SOX1, titin, recoverin
	blot A only	4/16	Line-blot (Ravo or Euroimmun), antigens: see above
Neuronal Cell Surface antibodies	Immunohistochemistry on rat brain + in-house CBA	2/16	In-house obtained slices from lightly fixed rat brain + in-house fixed (Euroimmun), or live CBA designed according to the staining pattern on tissue (10, 36)#
	In-house CBA	1/16	Live CBAs for specific antigens (38, 39)#
	Commercial CBA	13/16	Fixed CBA mosaic chip (Euroimmun); antigens: NMDAR, LGI1, CASPR2, AMPAR 1/2, GABA_B_R
AQP4 antibodies	In-house CBA	2/20	Live CBA, transfection with M23 AQP4 isoform
	Commercial CBA	17/20	Fixed CBA (Euroimmun), transfection with M23 AQP4 isoform
	Commercial ELISA	1/20	RSR Limited, no information on AQP4 isoform used
MOG antibodies	In-house CBA A	3/13	Live CBA, transfection with full-length MOG, total IgG secondary antibody, titration cut-off (1:160) (8, 9)#
	In-house CBA B	1/13	Live CBA, transfection with full-length MOG, IgG1 secondary antibody (7)#
	In-house CBA C	1/13	Live CBA, transfection with full-length MOG, total IgG secondary antibody, titration cut-off 1:160 + IgG1 secondary antibody (7, 9)#
	In-house live CBA D	2/13	Like CBA A, cytofluorimetric analysis (40)#
	Commercial CBA	6/13	Live CBA, transfection with full-length MOG, total IgG secondary antibody, titration cut-off (1:10)
MAG antibodies	Commercial ELISA	10/14	Bühlmann
	Immunohistochemistry	1/14	Commercial, Immco Diagnostics
	Immunohistochemistry+blot	1/14	Commercial, not specified
	Commercial blot	1/14	Ravo
	Commercial blot	1/14	Euroimmun
Antibodies to Gangliosides	In-house ELISA	5/15	In accordance with INCAT (41)
	Commercial blot	3/15	Line blot (Euroimmun)
	Commercial ELISA	4/15	Bühlmann
	Commercial blot	3/15	Dot blot (Generic Assay)
AChR antibodies	Commercial RIA	5/8	IBL International; RSR Limited
	Commercial ELISA	3/8	RSR Limited
MuSK antibodies	Commercial RIA	4/5	RSR Limited
	Commercial ELISA	1/5	RSR Limited

*NMDAR, N-methyl-D-aspartate receptor; LGI1, leucine rich glioma inactivated 1; CASPR2, contactin-associated protein-like 2; GABA_B_R, γ-aminobutyric acid B receptor; AMPAR, α-amino-3-hydroxy-5-methyl-4-isoxazolepropionic acid receptor; AQP4, aquaporin 4; ELISA, enzyme-linked immunosorbent assay; MOG, myelin oligodendrocyte glycoprotein; MAG, myelin associated glycoprotein; INCAT, Inflammatory Neuropathy Cause and Treatment; RIA, radioimmunosorbent assay; AChR, acetylcholine receptor; MuSK, muscle specific kinase*.

* *Single laboratories can use more than one test; CBA, cell-based assay; #In-house assays were performed according to published protocols, but adapted to each laboratory routine*.

Sample C2 [from a patient with definite NMDAR encephalitis (32)] was the only one providing conflicting results, as 9/14 laboratories failed to detect NMDAR antibodies. This sample tested at the coordinating center showed 1:200 positive titer using a TBA, and 1:10 positive titer using the commercial CBA (weak positivity) (42).

#### Conclusions

Discrepancies were mainly due to difficulties in detecting low titer NMDAR-Abs. This supports the message that, in autoimmune encephalitis, testing for both serum and CSF can increase diagnostic accuracy (42). Indeed, the paired CSF sample of the C2 control was positive at high titer.

### Intracellular Neuronal Antibodies

#### Background of the Assay

ICN-Abs target nuclear or cytoplasmic antigens, and associate with a wide range of neurological syndromes often occurring in association with a tumor (paraneoplastic neurological syndromes, PNS). Classic PNS include, among others, limbic encephalitis, paraneoplastic cerebellar degeneration, and subacute sensory neuronopathy (43, 44). Although their association with cancer is much rarer, GAD-Abs are often included in this group, and are associated with stiff person syndrome, epilepsy, or cerebellar ataxia, but also type-1 diabetes (45–47).

ICN-Abs are usually identified with screening TBAs on murine or primate cerebellum, followed by confirmatory SPAs (commercial line/dot blots). Blots include the most common antibody targets, with some differences on the panel according to the manufacturer (Table 2). Although blots can be more sensitive than TBA in rare cases (48), their use without TBAs can lead to false positive results, and is therefore discouraged (4, 49, 50). In-house CBAs have been used with selected antigens, such as CV2 and SOX1, showing a higher sensitivity compared to commercial blots (51, 52). GAD antibodies can be quantified using ELISAs, RIAs, and luciferase immunoprecipitation system (LIPS) (47, 53), which are more sensitive than TBAs and line/dot blots (4).

#### Results of AINI EQAS

Among the participating laboratories, the ICN-Ab detection was characterized by some heterogeneity of laboratory assays. Twelve/sixteen laboratories used a combination of TBAs and confirmatory SPAs, with two different commercial line blots (each used by six laboratories). Four laboratories did not perform a screening with TBA. No laboratory performed ELISAs, or LPAs for GAD antibodies.

Overall agreement for this scheme was “fair” (Fleiss' Kappa: 0.39, 95%CI: 0.3–0.49).

Sample O2 was wrongly identified as negative by 4/16 laboratories. One of the laboratories detected a compatible staining pattern with TBA, not confirmed on line blots, and the three remaining laboratories performed the line blot only. In addition, for the same sample 8/16 laboratories additionally reported a positivity for titin-Abs detected with line blots. The same reactivity was reported by 7/16 laboratories with the sample O3 (sent as negative). Titin-Abs are variably used in patients with myasthenia gravis (MG) as biomarker of thymoma (54). O2 and O3 patients did not show any clinical manifestation of MG, and had no thymoma.

#### Conclusions

The poor performances of many laboratories in this EQA scheme could have the following main reasons: (a) TBAs are mandatory screening tests (42), so that using only line/dot blots, based on recombinant proteins, can yield false positive and false negative results (49); (b) the recognition of particular ICN-Abs patterns on TBAs is challenging (42); (c) faint antibody reactivities on line/bot blots should be interpreted as negative results.

The introduction of the titin antigen in the commercial dot/line blots for ICN-Abs is questionable, as MG had been considered an “independent disease,” and thus excluded by the diagnostic criteria for PNS (42).

### Ganglioside Antibodies

#### Background of the Assay

Ganglioside-Abs are associated with a wide spectrum of inflammatory peripheral neuropathies (55). However, only few of them have actual diagnostic meaning and associate with well-defined clinical phenotypes. These include: (a) antibodies against a dialosyl epitope, a sequence contained in GD1b, GD3, GT1b, and GQ1b molecules in patients with a paraproteinemic neuropathy defined as CANOMAD (Chronic Ataxic Neuropathy, Ophthalmoplegia, IgM paraprotein, cold Agglutinin, Disialosyl antibodies) (56, 57); (b) GM1 IgM-Abs in patients with motor multifocal neuropathy with conduction blocks (MMN) (58); (c) GQ1b (with/without GT1a) IgG antibodies in patients with Fisher syndrome, a variant of Guillain-Barrè syndrome (GBS) with ophthalmoplegia and ataxic neuropathy (59, 60). GD1a, GM1a, GM1b, and GalNAc-GD1a-Abs (IgG isotype) characterize the acute motor axonal neuropathy (AMAN), and GM1 and GD1a-antibodies (IgG isotype) characterize acute motor and sensory axonal neuropathy (AMSAN), but these axonal forms of GBS are more common in Asia and Central and South America than in North America and Europe.

Thin layer chromatography is considered the gold standard, but it is often unavailable for routine diagnostics, for which available options include line/dot blots and ELISAs, with suboptimal performances (56). In order to improve standardization, in 1999 an ELISA for ganglioside-Abs has been proposed by an experts panel (INCAT-ELISA) (41), and is still considered a valid assay notwithstanding the documented inter-laboratory variability (61). Limiting the tests to the above-mentioned autoantibodies, and considering positive results only when high titers are detected represent useful recommendations for clinicians (61).

#### Results of AINI EQAS

Five/fifteen laboratories performed ELISAs according to the INCAT guidelines, 4/15 used commercial ELISAs, and 6/15 commercial blot from two different manufacturers (Table 2). The EQA scheme for ganglioside-Abs had the lowest agreement (Fleiss' kappa: 0.29, 95%CI: 0.21–0.36), and the lowest accuracy (median: 50; range: 25–100) within the EQA program. Twelve/fifteen laboratories performed suboptimally, showing an accuracy ≤ 50%. Sample P1 (from a patient with Fisher syndrome) was correctly reported as GQ1b-IgG positive by 14/15 laboratories, but three laboratories additionally identified other ganglioside-Abs, such as GM1-IgM, or GT1a IgG, which, however, can coexist with GQ1b-IgG. Sample P3 (from a patient with CANOMAD) was classified as positive for both GD1b and GQ1b-IgM. Only three laboratories showed agreement with the reference value. Four/fifteen laboratories reported only one of the two ganglioside-Abs, whilst four reported additional ganglioside-Abs, possibly compatible with the clinical syndrome (such as, GD1b-IgM), or unrelated (such as, sulfatide IgM). Similarly, in sample P4 (from a patient with MMN, sent as GM1-IgM-positive) 9/15 laboratories reported additional reactivities including GM2 and GD1b-IgM. The interpretation of this scheme thus needed the arbitrary setting up the category of “partially concordant” results, when antibody reactivities compatible with the established clinical phenotypes were reported in addition to the reference reactivities. However, the statistical analysis of this EQAS was calculated including “partially concordant” results into the category of “discordant” results.

#### Conclusions

This scheme was the most critical of our EQA program, likely due to the relatively high heterogeneity of the tests employed by the various laboratories, and the technical drawbacks that intrinsically affect the current methods for ganglioside-Abs testing (62). The poorest performances still remain even if the category of “partially concordant” joins that of “concordant” results.

### MAG Antibodies

#### Background of the Assay

MAG neuropathy is a rare disease typically associated with monoclonal IgM that recognize the glycoprotein (63). A slowly progressing neuropathy characterizes the disease (Distal acquired demyelinating symmetric neuropathy, DADS). MAG-Abs detection is preferentially performed with ELISA, which produces quantitative results useful for monitoring the disease. Other tests, including Western or line blot, and TBAs are available, but they show lower accuracy (63, 64).

#### Results of AINI EQAS

Despite the heterogeneity of the assays used (Table 2), all laboratories correctly identified MAG-Abs in the three reference samples, all from patients with DADS.

#### Conclusions

MAG scheme was not critical. However, among the laboratories that used the Bühlmann ELISA, large differences in quantitative values were detected, thus suggesting between-laboratory difference in performing the test.

### AChR and MuSK Antibodies

#### Background of the Assay

MG is an autoimmune disorder of the neuromuscular junction characterized by muscle fatigue and reduced endurance upon repetitive use (65, 66). AChR-Abs are highly specific for MG, and are found in 85–90% of patients with generalized MG and in 40–70% with ocular MG (66, 67). More recently discovered, MuSK-Abs are present in serum samples of about one third of AChR-Abs-negative MG patients (68, 69).

LPA, and particularly RIA, either “in-house” or commercially available, are considered the gold standard for both AChR- and MuSK-Ab detection (69). Recently, novel tests using CBAs have been implemented, showing high sensitivity in detecting AChR- and MuSK-Abs in LPA antibody-negative patients (70, 71). This advantage is likely linked to the antigen clustering at the cell surface, thus improving the binding of divalent low-affinity AChR-Abs. However, such tests are performed on live cells, and thus they are necessarily “in-house” and non-standardized. Alternatively, commercial ELISAs are available for the detection of both AChR- and MuSK-Abs, but their performances are inferior to those of RIAs (69).

#### Results of AINI EQAS

The number of laboratories participating to AChR- and MuSK-Ab schemes was limited (8 and 5, respectively). Three laboratories in the AChR-Ab scheme, and one in the MuSK-Ab scheme, used a recently released commercial ELISAs, whilst the remaining laboratories used the consolidated commercial RIAs. Accuracy was high, but one laboratory using the ELISA identified MuSK-Abs in a negative sample.

#### Conclusions

RIAs remain the gold standard for AChR- and MuSK-Ab detection. CBAs for their detection are showing promising preliminary results (38), and forthcoming EQA programs will evaluate their performances.

## General conclusions

The Holy Grail of precision medicine requires endless efforts toward the production of biomarker data for accurate stratifications of patients, and, to determine the best approach to prevent, diagnose, or treat diseases. These efforts are exploiting the impressive technological advancements to identify new biomarkers. On the other hand, the contribute of well-established biomarkers should not be overlooked.

The here reported data from the 2018 AINI EQA program depict a complex picture on how currently used neuroimmunology biomarkers work in real life. The evidence derives from a single EQA evaluation, but we found similar performances in our previous AINI EQA programs [personal communication].

Briefly, the neuroimmunology tests here evaluated can fall into three categories:

(a) standardized and robust commercial tests with substantial inter-laboratory agreement (MAG-Abs; AChR- and MuSK-Abs); (b) commercial and “in-house” tests with partial inter-laboratory agreement (AQP4-Abs, MOG-Abs, NS-Abs, ICN-Abs); (c) commercial and in-house tests with large inter-laboratory disagreement (OCBs, ganglioside-Abs).

The CBAs used for AQP4 and MOG-Ab detection are of relatively recent introduction. Both in-house and commercial tests seem to perform suboptimally in low-titer sample controls. Accordingly, a large multicenter comparison of various tests for AQP4-Abs suggests that technical accuracy improves when tests are carried out in specialist laboratories (18).

As a whole, technical inaccuracy and shortcomings in results interpretations are likely the main reasons underlying the suboptimal performance put in evidence by our EQA program for NS- and ICN-Abs too. However, there are two tests that carry well-known “structural” limits, namely the IEF for OCB detection (8), and ELISA, or dot/line blot tests for ganglioside-Abs (60), which are very difficult to overcome. As for OCBs, such limits were one of the main points supporting their exclusion from MS diagnostic criteria (72). Exploiting the expertise of specialized laboratory, with a centralization of OCB testing, could minimize the above-mentioned shortcomings. The limits of the available tests for ganglioside-Abs, once recognized, should lead to a consensus including experts and the main manufacturers, to find the best compromise on the best single method to use and on interpretative rules for positive results.

The commercial fixed CBA for MOG-Abs seemed to perform as well as the in-house live CBAs, but only three samples were tested, not allowing the due statistical evaluations. The in-house live CBA for MOG-Abs yielded better results than a fixed CBA in a three-center comparison study (29).

The main limitation of this study is the low number (3 or 4) of samples sent for each assay. On the other hand, high volumes of control samples from patients with a given disease, that are necessary when many centers are involved in EQA programs, are not easily obtainable, and evaluations on single assay performances should better imply high numbers of samples tested by a few selected centers.

In conclusion, our findings give clinicians a panorama of what they can expect when they ask for neuroimmunology tests. Although restricted to Italian and a few European laboratories, the data of this EQA program are indeed in line with other similar surveys promoted for single tests (18, 29, 50). It is conceivable that in countries where neuroimmunology diagnostics is centralized in laboratories with specific expertise the quality of the service could be higher. Further efforts for standardizations are still needed, as well as the promotion of EQA programs, which are fundamental even for expert laboratories.

## Data Availability Statement

The datasets generated for this study are available on request to the corresponding author.

## Author Contributions

MG analyzed the data, drafted the manuscript, and cooperated to the AINI-EQAP planning and coordination. DF drafted the manuscript and was the main planner and coordinator of the AINI EQAP. AU, FB, and FA contributed to plan the AINI-EQAP and revised the manuscript for intellectual content. SS and EZ contributed to sample preparation and shipping in the EQAP and to data analysis.

### Conflict of Interest

The authors declare that the research was conducted in the absence of any commercial or financial relationships that could be construed as a potential conflict of interest.
