# Supplementary material for: Autoantibody Diagnostics in Neuroimmunology: Experience From the 2018 Italian Neuroimmunology Association External Quality Assessment Program
Source: Front Neurol. 2020 Jan 14;10:1385. doi: 10.3389/fneur.2019.01385 (PMC6971200; doi:10.3389/fneur.2019.01385)
Supplement: Supplementary file 1 [file Table_1.DOCX]

Supplementary material

**Supplementary table 1: Participants to the 2018 AINI EQAP**

| Laboratory Number | Institution | Participants |
| --- | --- | --- |
| 1 | Neuroimmunology Laboratory, IRCCS Mondino Foundation, Pavia, Italy | Diego Franciotta, Matteo Gastaldi, Silvia Scaranzin, Elisabetta Zardini, Silvia Romagnolo |
| 2 | Neuroimmunology laboratory, UO Neurology IV  Laboratory of Clinical Pathology and Medical Genetics  Foundation IRCCS Istituto Neurologico Carlo Besta, Milan, Italy | Francesca Andreetta, Fulvio Baggi, Ornella Simoncini, Renato Mantegazza (Neurology IV)  Emilio Ciusani, Gaetano Bernardi (Laboratory of Clinical Pathology and Medical Genetic) |
| 3 | Clinical chemistry laboratory, Ca’Foncello Hospital, Treviso/Pediatric Institute “Città della Speranza”, Padova, Italy | Luigi Zuliani, Piera De Gaspari |
| 4 | Clinical Chemistry Laboratory, Vicenza Hospital, Vicenza, Italy | Elisabetta Galloni, Valentina De Riva |
| 5 | Clinical chemistry laboratory, ASST Monza, Monza/Milano-Bicocca University, Milan, Italy | Rinaldo Brivio, Vanna Elisabetta Minolfi  (Laboratorio Analisi of the ASST Monza), Guido Cavaletti |
| 6 | Neuroimmunology laboratory, Careggi hospital, Florence, Italy | Tiziana Biagioli, Luca Massacesi |
| 7 | Neurology Unit, Dept. of Neurosciences, Biomedicine and Movement Sciences, University of Verona, Italy | Sergio Ferrari, Sara Mariotto |
| 8 | Neuroimmunology Laboratory, Innsbruck, Austria | Kathrin Schanda, Markus Reindl |
| 9 | Clinical Pathology Laboratory, “Policlinico Santa Maria alle Scotte di Siena”, Siena | Alessandro Pini, Luisa Bracci |
| 10 | Neurochemistry Lab - Dept of Basic Medical Science, Neuroscience and Sense Organs -University of Bari-Aldo Moro, Bari, Italy | Maddalena Ruggieri, Antonio Frigeri |
| 11 | Dept. of Medical and Surgical Sciences, University of Foggia, Foggia, Italy / Dept. of Laboratory Diagnostics, Azienda Ospedali Riuniti, Foggia, Italy | Carlo Avolio, Michele Falcone |
| 12 | Neuroimmunology Laboratory, Nuffield Department of Clinical Neurosciences (NDCN), John Racliffe hospital, Oxford (UK) | Patrick Waters, Sarosh irani, Mark Woodhall, Leslie Jacobson |
| 13 | Neuroimmunology laboratory, Institut d'Investigacions Biomèdiques August Pi i Sunyer (IDIBAPS), Hospital Clinic, Barcelona, Spain | Maro Rhodes, Francesc Graus, Josep Dalmau, Marianna Spatola |
| 14 | Neuroimmunology Laboratory, Queen Elizabeth University Hospital, Glasgow, UK | Hugh Willison, Denise Marshall |
| 15 | Laboratorio di Neurobiologia Clinica e Diagnostica Liquor, Ospedale Santa Chiara, Pisa, , Italy | Andrea Bacci, Livia Pasquali |
| 16 | Lab Neurobiologia, palazzina Ottolenghi, AUO S Luigi, Orbassano, Italy | Arianna Sala, Antonio Bertolotto |
| 17 | Laboratorio Analisi Chimico Cliniche, ASST Papa Giovanni XXIII, Bergamo, Italy | Maria Grazia Alessio |
| 18 | Laboratorio di neurochimica, AZIENDA OSPEDALE POLICLINICO DI BARI , Bari, Italy | Rosaria Leante |
| 19 | Autoimmunità e Allergologia, Ospedale Maggiore, Bologna, Italy | Gaia Deleonardi |
| 20 | Lab. Centro SM, ASL8 - Ospedale Binaghi, Dip. Sanità Pubblica, Medicina Clinica e Molecolare, Cagliari, Italy | Gianna Costa |
| 21 | U.O.C. Patologia Clinica, Ospedale Garibaldi Centro, Catania, Italy | Diana Cinà |
| 22 | Centro SM, Clinica Neurologica, Policlinico SS Annunziata, Chieti, Italy | Giovanna De Luca |
| 23 | Lab Neurochimica, AOU Arcispedale S. Anna, Ferrara, Italy | Massimiliano Castellazzi |
| 24 | Laboratorio Centrale, Ospedale di Gallarate, Gallarate, Italy | Paola Pettini, Claudio Soldavini, Mauro Zaffaroni |
| 25 | Laboratorio Neuroimmunologia, Dipartimento di Neuroscienze, Riabilitazione, Oftalmologia, Genetica e Scienze Materno Infantili (DINOGMI), Genova, Italy | Debora Giunti, Elisabetta Capello, Claudio Panarese, Antonio Uccelli |
| 26 | Laboratorio Analisi, Ospedale San Raffaele, DIBIT2, Milano, Italy | Stefania Del Rosso, Massimo Locatelli |
| 27 | Laboratorio Neuroimmunologia, Nuovo Ospedale Civile S. Agostino Estense, Baggiovara Modena, Italy | Roberta Bedin, Diana Ferraro, Patrizia Sola |
| 28 | Sieroimmunologia e Allergologia, UOC Analisi Chimico Cliniche, Ospedale S. Stefano, Prato, Italy | Annalisa Azzurri |
| 29 | UOC Laboratorio Analisi, Ospedale Sant'Andrea, Roma, Italy | Maria Letizia Troccoli |
| 30 | Istituto di Patologia generale, Fondazione Policlinico A. Gemelli, Roma, Italy | Emanuela Bartoccioni, Mariapaola Marino, Raffaele Iorio |
| 31 | Lab Autoimmunità, Istituto Clinico Humanitas, Rozzano, Milano, Italy | Claudia Giannotta, Eduardo Nobile-Orazio |
| 32 | Laboratorio analisi SS Annunziata - settore Neurochimica e Neuroimmunologia, Azienda Ospedaliero-Universitaria di Sassari, Sassari, Italy | Giovanni Andrea Deiana |
| 33 | Klin.-immunologisches Labor, Lübeck, Germany | Bianca Teegen, Lars Komorowski, Prof. Dr. Stöcker |
| 34 | UO NEUROLOGIA, OSP. di TREVISO, Treviso, Italy | LUIGI ZULIANI |

**Supplementary table 2: Agreement and median accuracy in different EQA schemes**

| **Scheme** | **Fleiss's Kappa (95%CI)** | **Accuracy % median (range)** |
| --- | --- | --- |
| **MOG** | 0.71 (0.5-0.92) | 100 (100-33.3) |
| **NS** | 0.67 (0.54-0.80) | 66.7 (100-66.7) |
| **AQP4** | 0.66 (0.52-0.79) | 100 (50-100) |
| **MuSK** | 0.66 (0.02-1) | 100 (100-66.7) |
| **IEF-bands** | 0.51 (0.36-0.67) | 87.5 (62.5-100) |
| **IC-N** | 0.39 (0.3-0.49) | 66.7 (0-100) |
| **IEF-pattern** | 0.31 (0.20-0.42) | 50 (25-100) |
| **Gangliosides** | 0.29 (0.21-0.36) | 50 (25-100) |

CI: confidence interval; MOG: myelin oligodendrocyte glycoprotein; NS: neuronal surface; AQP4: aquaporin 4; MuSK: muscle specific kinase; IC-N: intracellular neuronal; IEF: isoelectric focusing.

**Supplementary figure 1: EQAP algorithm**


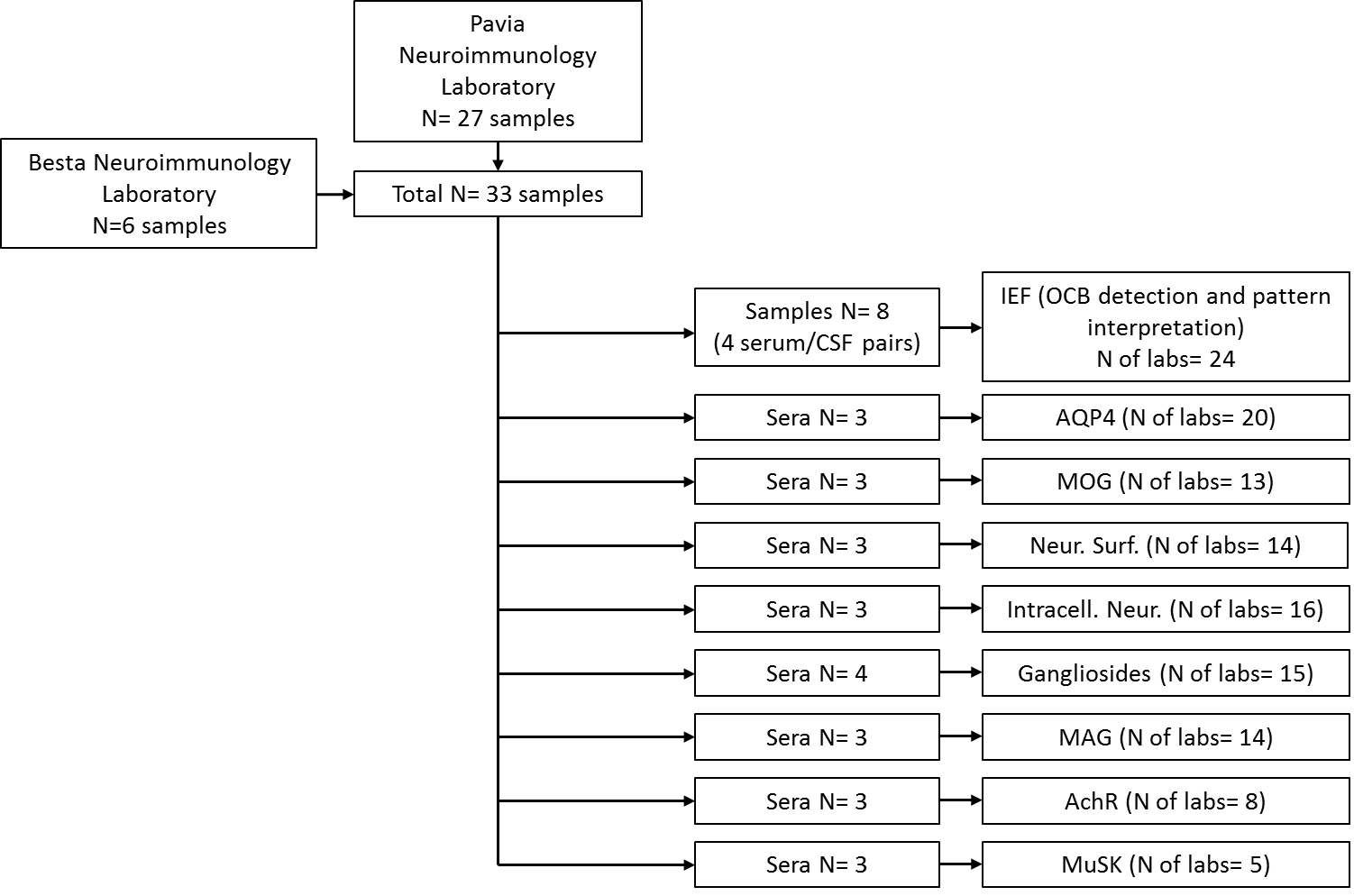


N: number; MOG: myelin oligodendrocyte glycoprotein; NS: neuronal surface; AQP4: aquaporin 4; MuSK: muscle specific kinase; AChR: acetylcholine receptor; IC-N: intracellular neuronal; IEF: isoelectric focusing.

**Supplementary figure 2: Samples management in the AINI-EQAP**


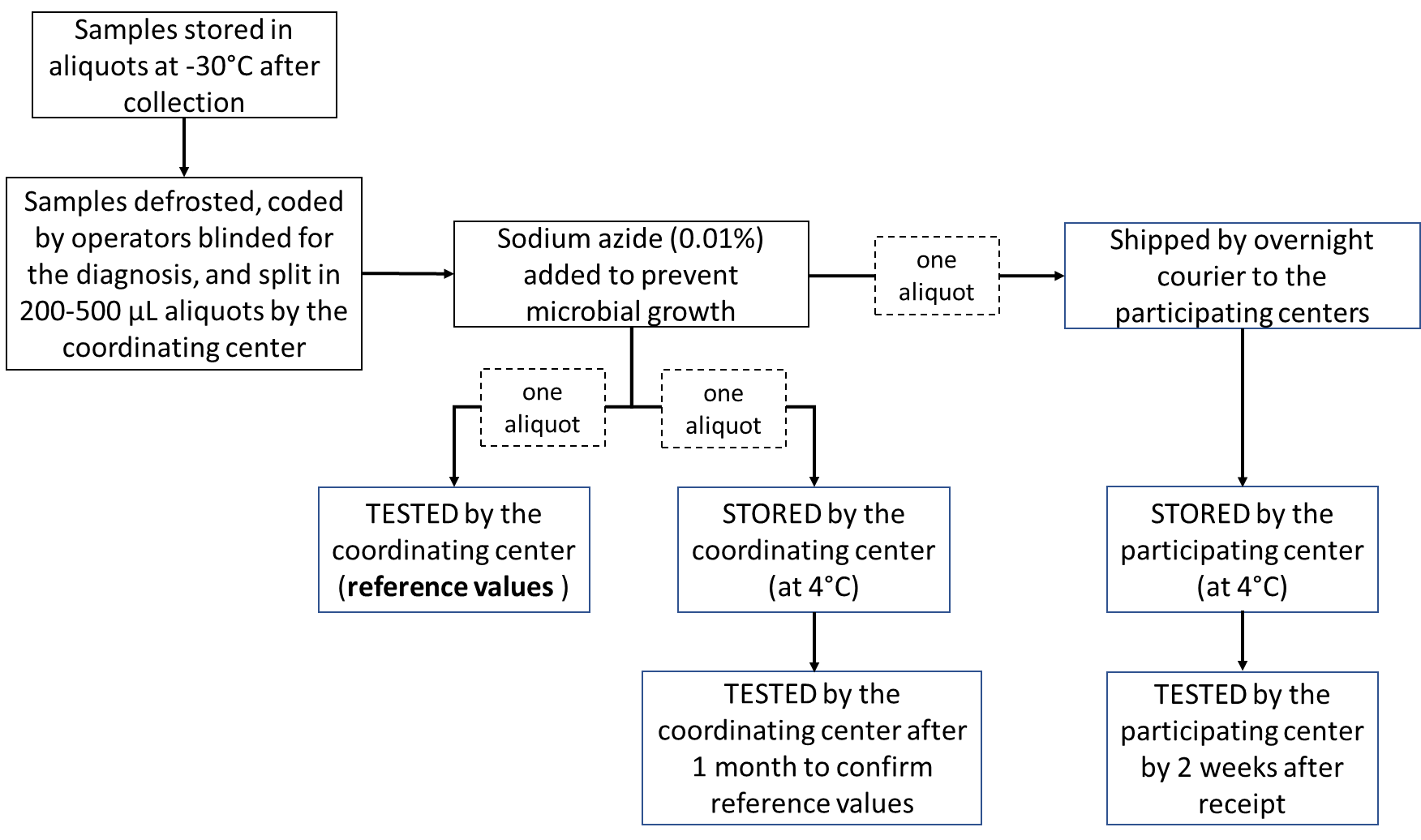


**Supplementary figure 3: EQA results in IEF scheme**

IEF: isoelectric focusing; 1: polyclonal pattern; 2: unique-to-cerebrospinal fluid (CSF) oligoclonal bands (OCB); 3: mixed pattern; 4: mirror pattern; 5 monoclonal gammopathy pattern.

**Supplementary figure 4: EQA results in AQP4, MOG, IC-N, NS, AChR and MuSK abs schemes**

MOG: myelin oligodendrocyte glycoprotein; NS: neuronal surface; AQP4: aquaporin 4; MuSK: muscle specific kinase; AChR: acetylcholine receptor; IC-N: intracellular neuronal; PCc: Purkinje cells cytoplasm; n.p.: not performed.

**Supplementary figure 5: EQA results in MAG and Ganglioside abs scheme**

Ganglio: gangliosides; MAG: myelin associated glycoprotein
